# Biological, phytochemical and molecular docking characteristics of *Laurus nobilis* L. fresh leaves essential oil from Palestine

**DOI:** 10.1186/s12906-024-04528-9

**Published:** 2024-06-08

**Authors:** Nidal Jaradat, Mohammed Hawash, Mohammed T. Qaoud, Nawaf Al-Maharik, Mohammad Qadi, Fatimah Hussein, Linda Issa, Ahmad Saleh, Laith Saleh, Ahmad Jadallah

**Affiliations:** 1 Department of Pharmacy, Faculty of Medicine and Health Sciences, An-Najah National University, Nablus, 00970 Palestine; 2 Faculty of Pharmacy, Cyprus International University, Nicosia, Cyprus; 3 Department of Chemistry, Faculty of Science, An-Najah National University, Nablus, 00970 Palestine; 4 Department of Biomedical Sciences, Faculty of Medicine and Health Sciences, An-Najah National University, Nablus, 00970 Palestine

**Keywords:** *Laurus nobilis*, Sweet Bay, Essential oil, Anticancer, Antioxidant, Antidiabetic, Anti-obesity, Antimicrobial, Molecular docking

## Abstract

**Background:**

The historical use of *Laurus nobilis* L., the plant is native to the Mediterranean region and has been cultivated for its aromatic leaves, which are used as a flavoring agent in cooking and for their potential therapeutic properties.

**Methods:**

The purpose of the current investigation was to characterize the essential oil composition of the fresh *L. nobilis* leaves from Palestine by using the gas chromatography-mass spectrometry (GC-MS) technique. DPPH (2,2-diphenyl-1-picrylhydrazyl), p-nitrophenyl butyrate, and 3,5-dinitro salicylic acid (DNSA) methods were employed to estimate the antioxidant, antiobesity, and antidiabetic effects of the essential oil. While MTS assay were used to evaluate their antiproliferative activities on panels of cell lines. Moreover, the docking studies were aided by the Prime MM GBSA method for estimating binding affinities.

**Results:**

The GC-MS investigation demonstrated that the fresh *L. nobilis* leaves essential oil has a variety of chemicals, about 31 different biochemicals were identified, and the major compounds were 1,8-cineole (48.54 ± 0.91%), terpinyl acetate (13.46 ± 0.34%), and α-terpinyl (3.84 ± 0.35%). Furthermore, the investigated oil demonstrated broad-spectrum antimicrobial activity against all tested bacterial and candidal strains and significantly inhibited the growth of MCF-7 cancerous cells more than the chemotherapeutic drug Doxorubicin. Furthermore, it contains robust DPPH free radicals, as well as porcine pancreatic α-amylase and lipase enzymes. Using the 1,8-cineole compound as the predominant biomolecule found in the *L. nobilis* essential oil, molecular docking studies were performed to confirm these observed fabulous results. The molecular docking simulations proposed that these recorded biological activities almost emanated from its high ability to form strong and effective hydrophobic interactions, this led to the getting of optimal fitting and interaction patterns within the binding sites of the applied crystallographic protein targets.

**Conclusion:**

The results of these experiments showed that the fresh *L. nobilis* leaves essential oil has outstanding pharmacological capabilities, making this oil a potential source of natural medications.

**Supplementary Information:**

The online version contains supplementary material available at 10.1186/s12906-024-04528-9.

## Background

Throughout history, the primary sources of medicines have originated from nature and have been utilized as remedies since ancient times, continuing to be valued and employed in contemporary healthcare [1, 2], the herbal medicine field has experienced exponential growth, gaining popularity in both developing and developed nations. This surge in popularity can be attributed to its natural origin and perceived lesser side effects compared to conventional pharmaceuticals [3]. Indeed, the utilization of plants in the cosmeceutical and pharmaceutical industries is extensive, with more than 50,000 plant species being employed for various purposes [4]. At least 3600 plant species are found in the Eastern region of the Mediterranean, encompassing Lebanon, Jordan, and Palestine [5]. The most economically affluent plant areas within the SP vascular plant taxa include the Gaza Strip (GS) with 1216 taxa, Jerusalem and Hebron Mountains (JHM) with 1235 taxa, and Nablus Mountains (NM) with 1126 taxa. Through agglomerative hierarchical clustering (AHC), the SP was categorized into two primary regions based on the presence of vascular plant taxa [6]. The varied topography of the country has facilitated the preservation of traditional knowledge concerning vegetable resources utilized by the local population as food. Despite this, there have been limited ethnobotanical studies on medicinal plants in certain areas of the country, and there is either no or very limited attention given to the exploration of wild edible plants [7].

Infectious diseases pose an escalating global menace, and the alarming rise of resistant microbial pathogens is a cause for concern [8]. Furthermore, antibiotic resistance is a global issue that significantly impacts human health [9]. Antimicrobial resistance presently leads to more than 7 million deaths each year, and this number is projected to rise to approximately 10 million deaths by the year 2050 [10].

Oxidative stress plays a critical role in the development of age-related diseases, encompassing conditions such as arthritis, diabetes, dementia, cancer, atherosclerosis, vascular disorders, obesity, osteoporosis, and metabolic syndromes [11]. Recent research findings indicate that natural compounds have the potential to decrease oxidative stress and enhance immune function [12, 13]. Oxidative stress arises from an imbalance in the body’s redox state, where the production of reactive oxygen species exceeds the natural defense mechanisms provided by antioxidants [14]. As a result, molecules with antioxidant activity play a crucial role in counteracting oxidative stress, contributing to the prevention of age-related diseases and the promotion of overall health [15].

According to surveys conducted by the World Health Organization (WHO), cancer stands as one of the leading causes of death worldwide [16]. Over the past few years, approximately 9 million deaths have been estimated annually due to this disease. Consequently, the discovery and development of novel anticancer agents are of utmost importance for the well-being of humanity [17]. Cancer stands as a prominent contributor to illness and death in Palestine, where lung cancer ranks as the predominant form among males, while breast cancer takes precedence among females [18]. In 2018, liver cancer was projected to be the sixth most frequently diagnosed cancer globally and the fourth leading cause of cancer-related deaths. It accounted for approximately 782,000 deaths and 800,000 new cases each year [19]. Since its inception nearly half a century ago, cancer chemotherapy has encountered significant challenges. The non-selective nature of conventional anticancer agents, causing damage not only to malignant cells but also to normal cells, particularly blood cells, has underscored the necessity for more targeted drugs. Another complication that emerged shortly after the initiation of cancer chemotherapy was the development of drug-resistant cancer cells. Consequently, there has been a growing interest in exploring potential anticancer agents from the plant life of various countries, often categorized in the market as “natural products” [20].

Diabetes is a prevalent chronic disease affecting populations worldwide [21]. In 2010, it was estimated that there were around 285 million adults with diabetes. The number of individuals affected by diabetes is projected to keep rising globally, primarily due to factors such as an aging population and overall population size growth [22]. Diabetes mellitus is classified as a metabolic disorder characterized by elevated blood sugar levels. There are two main types of diabetes: type I and type II. Type I diabetes is more prevalent in children and accounts for about 5–10% of all diabetes cases. In East Asia, the incidence rate is approximately one new case per 100,000 people per year [23]. In a prior investigation, 1883 individuals diagnosed with diabetes were interviewed. The majority of participants disclosed the use of herbs predominantly sourced from Palestine (98%), typically in their raw form, with a prevalent method being decoctions (44.1%). The top five herbal products frequently employed were *Trigonella berythea* (Fabaceae), *Rosmarinus officinalis* (Lamiaceae), *Olea europaea* (Oleaceae), *Teucrium capitatum* (Lamiaceae), and *Cinnamomum zeylanicum* (Lauraceae) [24].

Obesity and overweight have become significant public health issues on a global scale. In 2016, approximately 39% of men and 40% of women aged 18 and over, totaling nearly 2 billion adults, were classified as overweight. Additionally, 11% of men and 15% of women, totaling more than half a billion adults, were classified as obese worldwide. Furthermore, the prevalence of overweight and obesity has seen a substantial rise over the past four decades. This alarming trend highlights the urgent need for effective strategies to address and combat these growing health challenges [25].

*Laurus nobilis* L. (Sweet Bay) is an aromatic plant and evergreen tree that falls under the Lauraceae family. Renowned for its delightful aroma, it is one of the most commonly used culinary spices worldwide. This plant is cultivated and naturally found in Mediterranean countries like Turkey, Spain, Morocco, as well as other temperate and warm regions across the globe [26]. Besides its unique aroma, it is used as a food flavoring agent and to cure diseases worldwide [27]. As shown by prior research, *L. nobilis* has many possible uses [28, 29]. For example, some studies have shown promise for its use in treating rheumatic illnesses, cancer, epilepsy, gastrointestinal disorders, and a variety of infectious diseases; others have shown promise for its use as an antioxidant and the preservation of food [30].

In Traditional Arabic Palestinian Herbal Medicine (TAPHM), *L. nobilis* leaves are used to treat hemorrhoids, diarrhea, peptic ulcers, gastrointestinal disorders, jaundice, psoriasis, mouth ulcers, throat inflammations, urinary tract inflammations, bronchospasm, cold symptoms, chest pain, menstrual pain, and many other uses [31, 32]. Due to the wide range of traditional therapeutic applications of *L. nobilis* leaves, the present study aims to identify the phytochemical constituents of *L. nobilis* fresh leaf essential oil and evaluate its antimicrobial, anticancer, antioxidant, antidiabetic, and anti-obesity activities. Thus, it will afford knowledge for further work. Through continued research and exploration of *L. nobilis* and its potential medicinal properties, it is hoped that this plant could lead to the development of formulations with clinical applicability in treating various diseases, ultimately bringing about future clinical benefits.

## Methods

### Preparation and extraction of L. nobilis essential oil

The *L. nobilis* plant’s leaves were collected in the Nablus area of Palestine in June 2022. Dr. Nidal Jaradat, a pharmacognosist at Palestine’s An-Najah National University, characterized the investigated plant in the university’s Department of Pharmacy at the Faculty of Medicine and Health Sciences. A voucher specimen code was assigned to the preserved plant sample (Pharm-PCT-1366). The collected fresh green leaves were washed with running water several times and then chopped into small pieces. The *L. nobilis* plant’s essential oil was extracted in the manner that Jaradat et al. previously reported [33]. In brief, 1 Kg of fresh leaves were hydrodistilled in a Clevenger-style device for 3 h to get the essential oil, which was then dried by using Sodium sulfate. The average percentage of extracted essential oil production from the fresh plant sample was 2.16 ± 0.04%. Prior to use in the studies, the essential oil was kept in an amber flask at a temperature of 6 ◦C.

### GC-MS assessment

The identification of *L. nobilis* essential oil was performed using GC-MS techniques on a Perkin Elmer Clarus 500 gas chromatograph with a Perkin Elmer Clarus 560 mass spectrometer [34–36] and the full description of this method was provided in the supplementary file.

### Antimicrobial activity

The antimicrobial activity of *L. nobilis* essential oil was evaluated following a previously established broth microdilution method [37]. The antibacterial effect was tested against five common bacterial species obtained from the American Type Culture Collection (ATCC), including *Escherichia coli, Klebsiella pneumoniae, Proteus mirabilis, Pseudomonas aeruginosa*, and *Staphylococcus aureus*, with ATCC numbers 25,922, 13,883, 12,453, 9027, and 25,923, respectively. Additionally, a clinical isolate of Methicillin-resistant *Staphylococcus aureus* (MRSA), while anticandidal effect was assessed against *Candida albicans* (ATCC 90,028). Full description of this method was provided in the supplementary file. The used concentrations (ranging from 0.1 µg/mL to 50 µg/mL) of *L. nobilis* essential oil. To validate the method, Doxycycline and Ciprofloxacin were used as controls for antibacterial activity, while Miconazole served as a control for anticandidal activity [38, 39].

### Porcine pancreatic lipase inhibitory assay

To assess the anti-obesity activity of *L. nobilis* essential oil, a porcine pancreatic lipase inhibition assay was conducted. Orlistat, a known anti-obesity and anti-lipase drug, was used as a positive control. The porcine pancreatic lipase inhibitory method was based on the protocol described by Zheng et al. with slight modifications [40] five different concentrations were used (10, 50, 100, 500, and 700 µg/mL) and the full description of this method was provided in the supplementary file.

### α-amylase inhibition assay

The α-amylase inhibitory activity of *L. nobilis* essential oil was evaluated using the standard method reported by Nyambe-Silavwe et al. with slight modifications [41, 42] the concentration series of 10, 50, 70, 100, and 500 µg/mL was used and the full description of this method was provided in the supplementary file.

### Antioxidant assay

To assess the antioxidant potential of *L. nobilis* essential oil, a solution of the oil (1 mg/mL) in methanol was serially diluted with methanol to prepare concentrations of 2, 5, 10, 20, 30, 50, and 80 µg/mL. To evaluate the antioxidant half-maximal inhibitory concentration (IC_50_) of *L. nobilis* essential oil and Trolox, the BioDataFit-E1051 program [43, 44] was utilized. This allowed for a quantitative comparison of the antioxidant activities of both substances. However, the full description of this method was provided in the supplementary file.

### Cytotoxicity method

For the culture of breast cancer (MCF-7), skin tumor (B16-F1), and colorectal adenocarcinoma (Caco-2) tumor cells obtained from ATCC, Rockville, MD, USA, RPMI 1640 medium was utilized as the culture medium. The cells were treated with various concentrations (125, 250, 500, and 1000 µg/mL) of the tested essential oil, as well as with Doxorubicin, which served as a positive control [45, 46]. However, the full description of this method was provided in the supplementary file.

### Molecular docking studies

Out of the 31 biomolecules identified within the *L. nobilis* essential oil, the 1,8-cineole compound was selected to perform the molecular docking analysis stemming from its superior abundance (48.54%) and was found as the primary component. Molecular docking simulation was done to examine the interaction pattern and geometry of 1,8-cineole besides foreseeing its mechanism of action within the binding sites of crystallized protein structures that were selected properly related to the tested biological assays: including anti-microbial, anti-diabetic, anti-lipase, and the cytotoxic anti-proliferative activity. For the anti-microbial activity, the crystallized urease target protein of helicobacter pylori (PDB code: 1E9Y) was selected, while the cytochrome P450 14 alpha-sterol demethylase (CYP51) (PDB code: 1EA1) was retrieved to represent the anti-fungal activity. The crystallographic structures of human pancreatic alpha-amylase (PDB code: 4W93), triacylglycerol lipase enzyme (PDB code: 1ETH), and the *Homo sapiens* survivin protein were nominated to represent the anti-diabetic, anti-lipase, and the cytotoxic activity, respectively. All the applied 3D-crystalographic structures of that protein targets were retrieved from the RCSB Protein Data Bank (http://www.pdb.org, accessed on 22 March 2023).

Using the protein preparation wizards, subjected to the Maestro Schrödinger interface 12.3, the obtained crystallographic protein targets were prepared and refined following a multistep procedure [47, 48]. The preparation step involved adding hydrogen atoms, allocating the bond orders, eliminating the water molecules beyond 3Å from the hetero atom, and optimizing the formal charges. Also, the protein structures were energetically minimized at OPLS4 force field and the structural geometry and hydrogen bonds were upgraded. After that, using the receptor grid generation module, a receptor grid for each protein target was generated and the binding sides’ 3D dimensions were identified concerning their co-crystallized ligands.

To draw and prepare the chemical structures of 1,8-cineole and reference ligands, The 2D-sketcher and Ligprep modules were utilized, respectively [49]. This procedure included addressing the bond length and angles alongside their stereochemistries and ring conformations at lower energy states. Besides that, using Epik, chiralities, the possible ionization states, and tautomers were generated at pH (7.0 ± 2.0), and in the end, they minimized at the force field OPLS4.

The Extra-accuracy (XP) docking procedure was applied to dock the finally prepared ligands into the generated receptor grids. The obtained docking poses were assessed based on the Glide score which reflects the binding affinity of that docked ligand with the target protein receptor, the lower the binding energy, the higher and the binding affinity. In the end, the Protein–Ligand Interaction Profiler (PLIP) server was utilized to get a more detailed binding pattern for the top-scored pose of each docked ligand [50].

### Statistical analysis

The tests for *L. nobilis* essential oil were performed in triplicate, ensuring three independent measurements for each concentration and cell line. The results were expressed as means (±) standard deviation (SD), representing the average value along with the variability in the data. Statistical analysis was carried out, and a *p*-value less than 0.05 was considered statistically significant, as well as the *p*-values were calculated by using t-test function in the Microsoft excel software accordingly. This significance level indicates that any observed differences or effects in the results were unlikely to have occurred due to chance, thus providing confidence in the validity of the findings.

## Results and discussion

### Phytochemical characterization

**Fig. 1:**
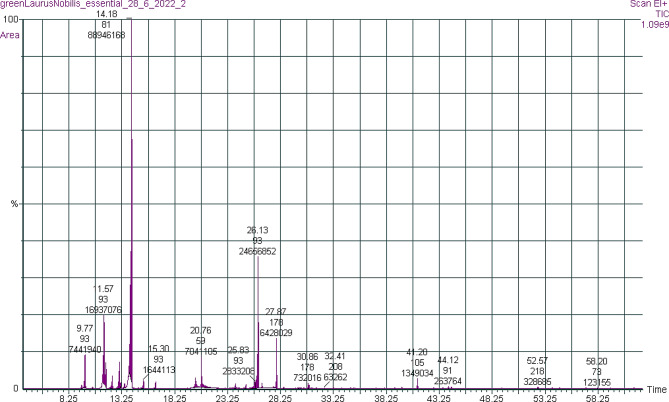
Gas Chromatography-Mass Spectroscopy (GCMS) chromatogram of *L. nobilis* essential oil

GC-MS analysis of *L. nobilis* essential oil enabled the identification and quantification of 31 different biochemicals from *L. nobilis* oil collected from Nablus /Palestine, representing 100% of the total extracted oil, as shown in Table 1; Fig. 1.

**Table 1:** Phytochemical constituents characterized by GC-MS of *L. nobilis* fresh leaves essential oil

Name	*R*.T	*R*.I	Area	% Area
α-Thujene	9.46	923	728,764	0.40 ± 0.01
α-Pinene	9.77	930	7,441,940	4.06 ± 0.2
Camphene	10.5	946	256,021	0.14 ± 0.02
Sabinene	11.57	969	16,937,076	9.24 ± 0.71
β-Pinene	11.74	973	5,154,842	2.81 ± 0.2
Myrcene	12.34	987	3,167,954	1.73 ± 0.03
α-Phellandrene	13.03	1002	5,912,282	3.23 ± 0.1
S-3-Carene	13.14	1005	1,214,502	0.66 ± 0.01
α-Terpinene	13.51	1013	899,935	0.49 ± 0.01
1,8-Cineole	14.18	1029	88,946,168	48.54 ± 0.91
γ-Terpinene	15.3	1055	1,644,113	0.90 ± 0.01
Terpinolene	16.44	1082	984,237	0.54 ± 0.01
Terpinen-4-ol	20.2	1178	3,756,729	2.05 ± 0.21
α-Terpinyl	20.75	1192	7,041,105	3.84 ± 0.35
Bornyl acetete	23.98	1281	623,768	0.34 ± 0.02
Isoverbanol acetate	24.99	1309	617,110	0.34 ± 0.41
Carvyl acetate, cis-	25.83	1334	2,833,208	1.55 ± 0.10
Terpinyl acetate	26.13	1343	24,656,852	13.46 ± 0.34
Neryl acetate	26.49	1354	718,065	0.39 ± 0.02
Isoeugenol methyl ether	27.57	1387	210,213	0.11 ± 0.03
Methyl eugenol	27.87	1396	6,428,029	3.51 ± 0.11
β-Caryophyllene	28.55	1417	217,921	0.12 ± 0.01
tran-methyl isoeugenol	30.86	1491	732,016	0.40 ± 0.01
Carvotanacetone	37.28	1716	33,633	0.02 ± 0.01
8-α-11-Elmodiol	38.08	1746	53,830	0.03 ± 0.01
β-Bisabolenol	39.03	1782	61,084	0.03 ± 0.01
Pentadecanoic acid	41.2	1867	1,349,034	0.74 ± 0.04
Methyl hexadecanoate	42.71	1926	52,902	0.03 ± 0.01
α-Chenopodiol-6-acetate	43.6	1964	77,676	0.04 ± 0.01
Ferula Lactone	44.12	1986	263,764	0.14 ± 0.02
Methyl tetradecanoate	44.39	1997	210,004	0.11 ± 0.01
SUM			183,224,777	100
**Phytochemical fractions**				
Hydrocarbon monoterpene				24.20
Oxygenated monoterpenoids				74.55
Hydrocarbon sesquiterpene				0.12
Oxygenated sesquiterpenoids				0.25
Others				0.88
Total				100.00

The primary components were as follows: 1,8-cineole (48.54 ± 0.91%), terpinyl acetate (13.46 ± 0.34%), and α-terpinyl (3.84 ± 0.35%). The analysis of *L. nobilis* essential oil composition revealed a significant percentage of the monoterpene fraction (99.62%), dominated by hydrocarbon monoterpenes and oxygenated monoterpenoids representing 98.75% of the total essential oil fractions.

Oxygenated monoterpenoids, on the other hand, were the largest group, accounting for 74.55% of the essential oil, with 1,8-cineole (48.54 ± 0.91%), terpinyl acetete (13.46 ± 0.34%) and α-terpineol (3.84 ± 0.35%) being the main components. Sesquiterpenes and sesquiterpenoids represented 0.37% of the oil, the hydrocarbons 0.12% (β-caryophelene 0.12%), and the oxygenated compounds 0.25% (0.03% of 8-α-11-elmadiol, 0.04% of α-chenopoliol-6-acetate, 0.03% of β-bisabolenol and 0.14% of ferula lactone.

According to El-Sawi et al.‘s investigation, the major constituents of *L. nobilis* essential oil from Egypt were 1,8-cineole (50.38%), α-terpenyl acetate (19.97%), and 4-trepinol (6.48%) [51]. Similarly, the main components of *L. nobilis* essential oil from Italy were found to be 1,8-cineole (31.9%), sabinene (12.2%), and linalool (10.2%) [52]. Moreover, the abundant molecules identified in the *L. nobilis* essential oil from Morocco were 1,8-cineole (52.43%), α-terpinyl acetate (8.96%), and sabinene (6.13%) [53].

### Antibacterial and anticandidal activities

Table 2 depicts the antimicrobial activity of *L. nobilis* essential oil against Gram-negative and Gram-positive bacterial and *candiada* species. The results revealed that *L. nobilis* essential oil has potential antibacterial and anticandidal activities against all the screened gram-positive, gram-negative and *candida* species.

**Table 2:** Antimicrobial MIC (µg/mL) of *Laurus nobilis* essential oil and positive controls

	Bacteria						Fungus
	Gram-Positives		Gram-Negatives				Yeast
Microbe	**MRSA**	***S .aureus***	***E. coli***	***K. pneumoniae***	***P. mirabilis***	***P. aeruginosa***	***C. albicans***
*L. nobilis* EO	50 ± 0	50 ± 0	50 ± 0	50 ± 0	12.5 ± 0	50 ± 0	0.39 ± 0
Miconazole	-	-	-	-	-	-	0.39 ± 0
Doxycycline	50 ± 0	0.16 ± 0.044	1.56 ± 0	1.3 ± 0.45	0.65 ± 0.22	12.5 ± 0	-
Ciprofloxacin	25 ± 0	1.56 ± 0	0.02 ± 0.01	0.04 ± 0.01	0.032 ± 0.01	0.32 ± 0.1	-

The *L. nobilis* essential oil demonstrated the highest antibacterial activity against *P. mirabilis*, with a MIC value of 12.5 µg/mL. Furthermore, it exhibited potential anti-MRSA activity, with MIC values of 50, 25, and 50 µg/mL compared to Doxycycline and Ciprofloxacin antibiotics, respectively. In addition, the *L. nobilis* essential oil showed strong activity against *C. albicans*, with the same MIC value (0.39 µg/mL) as the commercial anti-yeast drug Miconazole.

These findings surpass previous reports that indicated *L. nobilis* essential oil’s potent antibacterial activity against *S. aureus, E. coli*, and *P. aeruginosa*, with MICs of 0.4, 0.8, and 0.4 μm/mL, respectively [52].

Comparing our results with older studies, it should be noted that *L. nobilis* essential oil from Nablus area of Palestine exhibited greater potential as an anticandidal agent compared to *L. nobilis* essential oil from Brazil, with a MIC value of 250 µg/mL, while *L. nobilis* essential oil from Palestine had MIC and MLC values of 0.39 µg/mL [30]. The present study’s outcomes are consistent with previous research that reported the antimicrobial potency of *L. nobilis* essential oil from various regions [29, 53, 54].

### DPPH free radicals, α-amylase and lipase inhibitory activities

Cancer, infertility, renal dysfunction, hepatic disorders, sleep problems, asthma, diabetes, and cardiovascular diseases are just some of the ways in which obesity reduces quality of life. Besides, obesity, oxidative stress, diabetes, cancer, inflammatory and infectious conditions are strongly correlated. Moreover, each disease listed can directly cause other diseases [55, 56].

Therefore, the current investigation determined to screen the effect of *L. nobilis* essential oil on DPPH free radical to assess its antioxidant effect, on porcine pancreatic lipase enzyme to evaluate its anti-obesity effect and on the pancreatic α-amylase enzyme to assess its anti-diabetic effect.

Table 3 depicts that the fresh *L. nobilis* essential oil has potent DPPH free radicals scavenging activity compared with vitamin E analog Trolox, which is considered the most powerful agent against free radicals. Actually, the fresh *L. nobilis* essential oil scavenged DPPH free radicals with an IC_50_ dose of 10.9 ± 0.38 µg/mL compared with Trolox, which has an IC_50_ dose of 2.88 ± 0.57 µg/mL.

A study established by El-Sawi et al. [51] reported that *L. nobilis* essential oil has DPPH free radical scavenging activity with an IC_50_ value of 0.52 mg/mL.

Moreover, the fresh leaves of *L. nobilis* essential oil has potent α-amylase inhibitory activity compared with the commercial antidiabetic drug Acarbose and the tested samples suppressed α-amylase enzyme with IC_50_ doses of 54.9 ± 0.93 and 28.18 ± 1.22 µg/mL, respectively.

These outcomes agreed with Başak and Candan investigation, which reported that the dried leaves of *L. nobilis* essential oil have potent α-amylase inhibitory activity with an IC_50_ dose of 42.12 ± 2.36 µg/mL [57]. Additionally, a study conducted by Al-Mijalli et al. found that the dried *L. nobilis* leaves essential oil had a potent α-amylase inhibitory activity with an IC_50_ dose of 42.51 ± 0.012 µg/mL compared with Acarbose which had an α-amylase inhibitory activity with an IC_50_ amount of 32.14 ± 0.016 µg/mL [58].

Besides, the fresh *L. nobilis* essential oil has potent lipase enzyme inhibitory activity compared with the commercial anti-obesity drug Orlistat. The tested samples suppressed porcine pancreatic lipase enzyme with IC_50_ doses of 66.07 ± 0.72 and 12.88 ± 0.94 µg/mL, respectively. These results with previously reported ones as in the Al-Mijalli et al. investigation, which documented the strong antilipase effect of the dried *L. nobilis* leaves essential oil with an IC_50_ dose of 21.23 ± 0.021 µg/mL compared with Orlistat (IC_50_ = 14.12 ± 0.023 µg/mL) [58].

**Table 3:** DPPH free radicals, porcine pancreatic α-amylase, and lipase inhibitory activities by fresh *Laurus nobilis* essential oil IC_50_ (µg/mL)

Samples	Fresh Laurus nobilis essential oil IC_50_ (µg/mL), ±SD
**Antioxidant activity**	10.9 ± 0.38
**Trolox**	2.88 ± 0.57
**α-Amylase inhibitory activity**	54.9 ± 0.93
**Acarbose**	28.18 ± 1.22
**Anti-lipase activity**	66.07 ± 0.72
**Orlistat**	12.88 ± 0.94

*P-value* < 0.05

Indeed, the phytochemical screening of the fresh leaves of *L. nobilis* essential oil revealed the presence of high contents of oxygenated monoterpenoid (74.55%). Monoterpenes have been found to enter the bloodstream and function as therapeutic molecules, as well as exerting a number of positive effects on people. Studies have demonstrated that natural monoterpenes and their synthetic derivatives possess various pharmacological properties, such as antispasmodic, antihistaminic, anti-inflammatory, local anesthetic, anti-aggregation, antiarrhythmic, anticancer, antioxidant, antifungal, antiviral, antibacterial, and antinociceptive activities [59].

### Cytotoxic and antiproliferative activity

The *L. nobilis* essential oil was tested against various cancer cell lines, including breast cancer (MCF-7), skin tumor (B16-F1), and colorectal adenocarcinoma (Caco-2) tumor cells. The results show that inhibition activity varies depending on the cancer cell line type. The results illustrate potent activity against MCF-7 cancer cells. The *L. nobilis* essential oil strongly suppressed the growth of MCF-7 tumor cells more than the powerful chemotherapeutic drug Doxorubicin with IC_50_ doses of 127.69 ± 2.05 and 324.12 ± 3.5 µg/mL, respectively. The detailed IC_50_ results are shown in Table 4.

**Table 4:** IC_50_ (µg/mL) doses of fresh *Laurus nobilis* essential oil against breast (MCF-7), skin (B16-F1), and colorectal (Caco-2) tumors cells compared with Doxorubicin (DOX)

Cancer cells	Laurus nobilis essential oil	DOX
**MCF-7**	127.69 ± 2.05	324.12 ± 3.5
**CaCo-2**	99.08 ± 1.78	>>0.05
**B16F1**	324.12 ± 3.5	>>0.05

*P-value* < 0.05

**Fig. 2:**
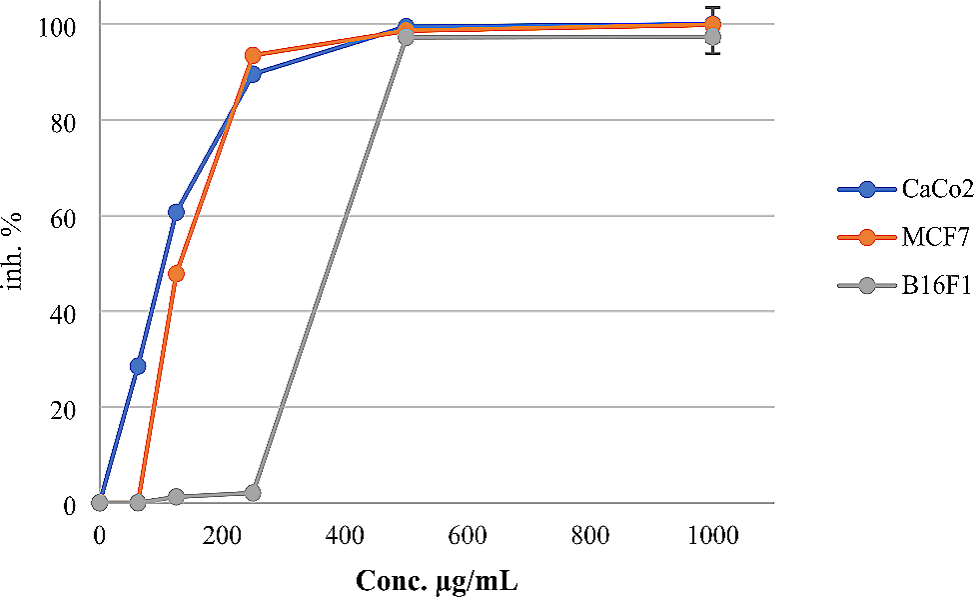
Percentage inhibition of cancer cell lines by *Laurus nobilis* essential oil at the concentration range of 0–1 mg/mL

The cytotoxicity results demonstrated potential inhibition activity of *L. nobilis* essential oil against Caco-2, MCF-7, and B16-F1 tested cancer cells. Moreover, a higher concentration of the plant oil (1 mg/mL) caused inhibition value of 99.99 ± 1.25, 99.88 ± 0.75, and 97.34 ± 1.01%, respectively (Fig. 2).

**Fig. 3:**
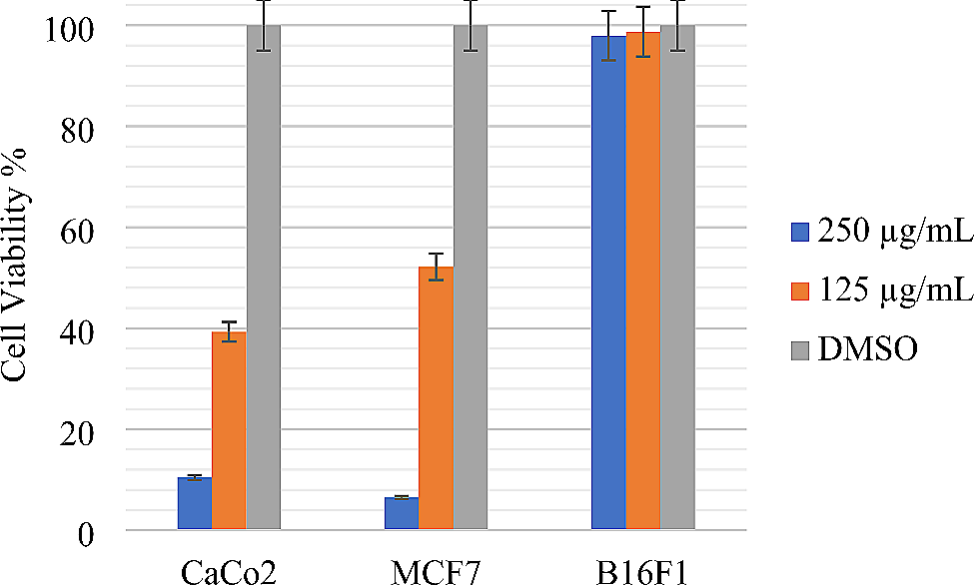
The Caco-2, MCF-7, and B16-F1 cell cancer viability treated with *Laurus nobilis* essential oil

In addition, at a concentration of 125 µg/mL of *L. nobilis* essential oil, the Caco-2, MCF-7, and B16-F1 cell viability decreased by 39.28, 52.15, and 98.75%, respectively, as demonstrated in Fig. 3.

### Molecular docking studies

**Fig. 4:**
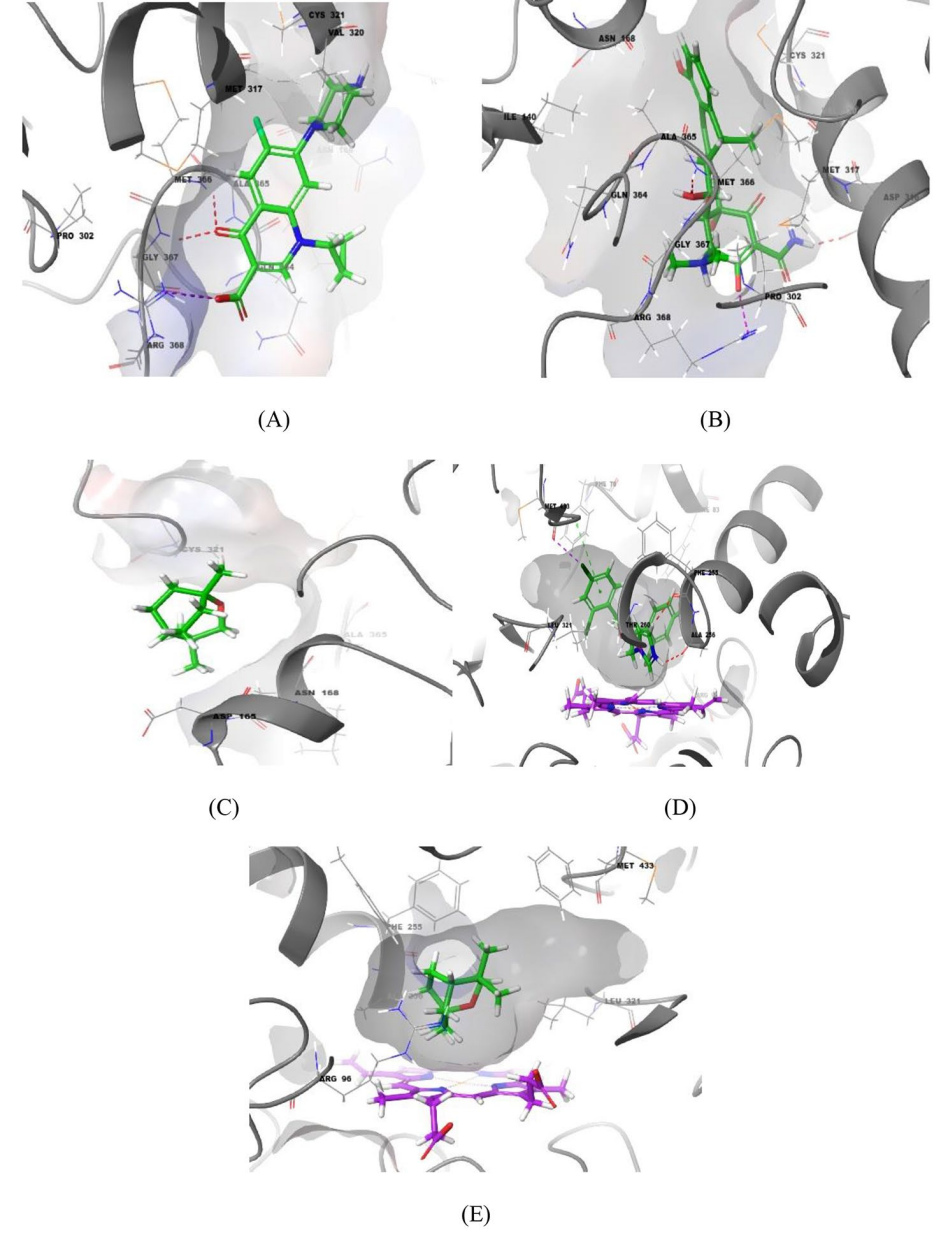
The molecular docking simulations of ciprofloxacin (**A**), doxycycline (**B**), and α-cineole (**C**) within the urease enzyme of the helicobacter pylori bacterial strain (PDB ID 1E9Y) shown as crystallized structure. Figures (**D**) and (**E**) represent the molecular docking simulations of miconazole and α-cineole, respectively, within the Cytochrome P450 14-alpha-sterol demethylase (CYP51) of C. albicans (PDB ID 1AE1). The hydrogen bonds and salt bridges are represented by dashed red and purple colors while the π-cationic interactions are represented by green colors

**Fig. 5:**
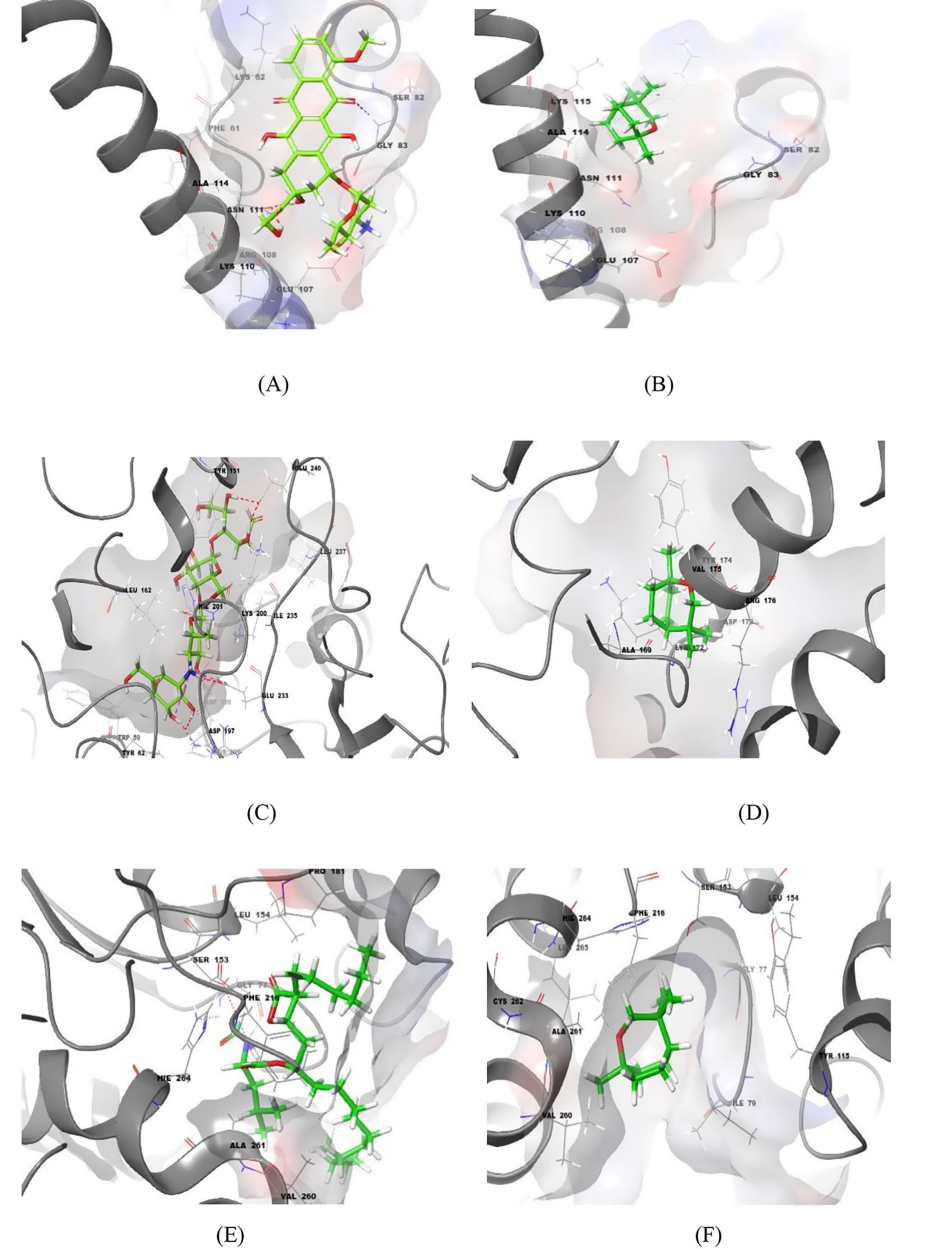
(**A**) and (**B**) demonstrate the molecular docking simulations of doxorubicin and α-cineole, respectively, within the binding site of the apoptotic inhibitor survivin (PDB ID 1E31) while figures (**C**) and (**D**) demonstrate the molecular docking simulations of the ligands acarbose and α-cineole, respectively, within the human pancreatic alpha-amylase (PDB ID 4W93). Figures (**E**) and (**F**) explain the docking simulation of the anti-diabetic agents orlistat and α-cineole within the binding site of lipase crystal structure (PDB ID 1ETH). The hydrogen bonds and salt bridges are represented by dashed red and purple colors

The performed *in-vitro* biological assays strongly indicated that the *L. nobilis* essential oil has a wide range of biological activities and experimentally showed potent anti-bacterial, anti-candidal, anti-diabetic, anti-lipase, and cytotoxic effects. As the 1,8-cineole compound was identified as the master biomolecule and found as the predominant (48.54%) over the other component within the *L. nobilis* essential oil, the 1,8-cineole compound was singled out to represent the oil extract. In the present study, *in silico* molecular docking simulations were performed on a set of crystallized protein targets to validate the reported experimental outcomes. This worthy approach aims to justify the interaction pattern and binding geometry of the docked ligands inside the binding site of that targetted protein receptor. All the docking simulations are shown in Figs. 4 and 5 while the docking scores obtained are summarized in Table 5.

Based on the observed potent anti-microbial activity of the *L. nobilis* essential oil, especially against the urease-splitting bacteria *P. mirabilis*, the 1,8-cineole, ciprofloxacin, and doxycycline were docked to the helicobacter pylori urease enzyme (PDB code: 1E9Y) to explicate the recorded activity alongside investigating their bioactive conformations and fitness docking scores within the binding site. The docking simulations of ciprofloxacin, doxycycline, and 1,8-cineole compounds are shown in Fig. 4-A, B, and C, respectively, and found to fit optimally within the urease binding pocket as inferred by the binding energy scores that ranged from − 6.976 K_cal/mol_ recorded for ciprofloxacin as the most potent compound to -4.392 k_cal/mol_ for the 1,8-cineole compound. This magical potency of ciprofloxacin against the *P. mirabilis* bacterial strain is rooted from the ideal fitting of its bioactive conformation within the binding pocket. It showed the formation of two hydrogen bonds with MET-366 and GLY-367 and one salt bridge between the carboxylic acid side chain and ARG-368 as a spellbinding interaction. Also, it was found within the binding range of GLN-364 and formed a new booster hydrophobic interaction. The docking simulation of doxycycline showed the formation of a pretty binding pattern within the binding site and properly formed two hydrogen bonds with ASP-316 and ALA-365 and one meager salt bridge with ARG-368. The 1,8-cineole compound, as a hydrophobic compound, formed mainly hydrophobic interactions and its biological activity almost arose from these valuable interactions. Analysis of the interaction profile using the PLIP server led to finding that 1,8-cineole compound successfully formed two hydrophobic interactions with ASP-165 and ASN-168 amino acids.

Aiming to illustrate the recorded biological anti-candidal activity of *L. nobilis* essential oil, molecular docking studies were performed for the1,8-cineole and miconazole compound, as a reference drug. Thus, their binding modes and fitting conformations were investigated within the binding site of Cytochrome P450-14-alpha-sterol demethylase target protein (PDB code: 1EA1), an administrative fungal enzyme and plays an essential role in sterol biosynthesis. The docking simulations of 1,8-cineole and miconazole compounds, as shown in Fig. 4-D and E, respectively, revealed their ideal fitting and they occupied the binding pocket optimally. The *in silico* binding pattern of miconazole showed the formation of hydrogen and halogen bonds with PHE-255, ALA-256, and MET-433 residues. Additionally, the dichloro phenyl group was located within the binding zone of PHE-78 residue and successfully formed π-π stacking interaction. On the other hand, the hydrophobic interactions seem to be the leading interactions of 1,8-cineole compound, as previously mentioned, that pushed the affinity toward fungal cells so could disrupt their vital biosynthesis pathways. Analyzing of the interaction pattern of 1,8-cineole showed the formation of many hydrophobic interactions with the surrounding residues such as PHE-255 and LEU-321.

As the cytotoxicity and anti-proliferative assay demonstrated a potential activity of *L. nobilis* against the tested cancer cell lines, especially against the MCF-7 cell line, the molecular docking approach was applied to confirm this observed activity alongside investigating the binding mode of the 1,8-cineole compound and doxorubicin, as a positive control drug. Regarding the resistance mechanisms involved in apoptotic cell death, so the efficiency of therapeutics decreases, the over-expression of Inhibitor of Apoptotic Proteins (IAP) is considered one of these critical mechanisms [60]. The apoptotic pathway could be blocked by many IAP members such as the survivin protein which cause a direct inhibition of the caspases proteins [61]. Taking into account that the MCF-7 cell line involves high expression levels of survivin, the crystallographic structure of survivin (PDB code: 1E31) was selected to be utilized for the docking studies [62]. Here, the doxorubicin drug, as a reference compound, was docked beside the 1,8-cineole compound for the binding site of the survivin target protein and the binding modes are illustrated in Fig. 5-A and B, respectively. The doxorubicin showed an ideal binding mode with optimal fitting to the binding site and showed the formation of four hydrogen bonds with ASN-111, GLU-107, and GLY-83 in addition to a salt bridge with GLU-107. Also, it formed one hydrophobic interaction with LYS-62. With respect to the 1,8-cineole, the hydrophobic interactions are also here observed as the dominant interactions and the lead source of its cytotoxic potency. Analysis of its obtained pose utilizing the PLIP server showed the formation of five favorable hydrophobic bonds with the surrounding residues LYS-62, ASN-111, ALA-114, and LYS-115.

Besides that, molecular docking studies were applied to confirm the anti-diabetic activity using the crystallographic protein structure of the human pancreatic alpha-amylase (PDB code: 4W93). The acarbose structure, as a reference anti-diabetic drug, and 1,8-cineole were docked to the binding pocket of the target protein and the docking simulations are shown in Fig. 5-C and D, respectively. The acarbose compound showed an advanced binding behavior through forming twelve hydrogen bonds that mainly worked in enhancing the affinity of acarbose to the binding site, referred to the superior binding energy value obtained which equaled − 14.469 k_cal/mol_. On the other side, the binding affinity of the 1,8-cineole compound, here again, stemmed from its hydrophobicity and showed the forming of many favorable hydrophobic interactions with ALA-169, LYS-172, ASP-173, TYR-174, VAL-157, and ARG-176.

Finally, the crystallographic structure of lipase (PDB code: 1ETH) was used as a target protein to be integrated for the molecular docking studies aiming to confirm the anti-lipase activity recorded to *L. nobilis* essential oil. The orlistat (as a reference) and 1,8-cineole ligands were docked to the binding site and the obtained docking simulations are represented in Fig. 5-E and F, respectively. As shown, the orlistat chimed optimally with the binding site geometry and revealed an ideal bioactive conformation involving the formation of two hydrogen bonds with GLY-77 and SER-153 residues. Additionally, it formed one salt bridge with HIS-264 alongside many hydrophobic interactions with the surrounding residues such as ILE-79, TYR-115, PRO-181, PHE-216, and VAL-260. With respect to the docking simulation of the 1,8-cineole compound, it also here confirmed the key role of hydrophobic interactions in boosting the ligand affinity to the tested biological targets. Analyzing the 1,8-cineole pose within the lipase binding pocket showed the formation of five hydrophobic interactions with the surrounding ILE-79, VAL-260, ALA-261, and LEU-265 residues.

**Table 5:** The docking scores of 1,8-cineole and the reference ligands within the protein targets utilized for the molecular docking studies

		Pharmacological targets (PDB ID codes)					
		*P. mirabilis* (1E9Y)	Breast Cancer cell lines (1E31)	Fungal Infections (1EA1)	Pancreatic Lipase (1ETH)	α-Amylase (3W93)	
Ligands	**Doxycycline**	-5.086	-	-	-	-	Docking scores (Kcal/mol)
	**Ciprofloxacin**	-6.976	-	-	-	-	
	**Doxorubicin**	-	-6.715	-	-	-	
	**Miconazole**			-7.460			
	**Orlistat**				-7.772		
	**Acarbose**					-14.469	
	**1,8-Cineole**	-4.392	-3.564	-5.551	-4.708	-5.864	

## Conclusions

Here, we currently report the phytochemical characterization of chemical components and some biological activities of the fresh *L. nobilis* leaves essential oil for the first time in Palestine. The GC-MS analysis showed a diversity of volatile molecules in the essential oil. The results showed potent antioxidant, antidiabetic, and anti-obesity potentials. Also showed that the investigated oil has antimicrobial effects against all the screened microbial species. Besides, with very low essential oil concentrations, it inhibits the growth of the MCF-7 tumorous cells strongly. Applying the molecular docking approach showed the master capability of the 1,8-cineole compound, the prevalent biomolecule found within the *L. nobilis* essential oil, to form many favorable hydrophobic interactions within the binding sites of the applied protein targets. As a result, it’s supposed that these observed wide-range biological activities are almost driven by these formed effective interactions. The 1,8-cineole compound as a simple, small, and hydrophobic structure alongside its high ability to form strong hydrophobic interactions, which have low energy penalties, all together participated mainly in enhancing its biological potency against the tested biological targets. These tests’ findings demonstrated that the fresh *L. nobilis* leaves essential oil has exceptional pharmacological characteristics and point it a potential source of natural medicines.

### Electronic supplementary material

Below is the link to the electronic supplementary material.

Supplementary Material 1

## Data Availability

All data generated or analyzed during this study are included in this published article (and its supplementary information files).

## Abbreviations

GC-MS: Gas chromatography–mass spectrometry
SP: State of Palestine
MRSA: Methicillin-resistant Staphylococcus aureus
MCF-7: Breast cancer
Hep 3B: Hepatocellular carcinoma
3D: Three dimensional
PKC: Protein kinase C
NADPH: Nicotinamide adenine dinucleotide phosphate hydrogen
WHO: World Health Organization
NCDs: Noncommunicable diseases
NCI: The national cancer institute
IC_50_: The half maximal inhibitory concentration
DPPH: 2,2-Diphenyl-1-picrylhydrazyl
UV: Ultraviolet
ATCC: American Type Culture Collection
DMSO: Dimethyl sulfoxide
RPMI: Roswell Park Memorial Institute
MIC: Minimum inhibitory concentration
AB: Absorbance of the blank
Ats: Recorded absorbance of the tested sample solution
DNSA: Dinitrosalicylic acid
PNPG: 4-Nitrophenyl-β-D-glucopyranoside
HEK: Human epithelial kidney
MTS: 3-(4,5-Dimethylthiazol-2-yl)-5-(3-carboxymethoxyphenyl)-2-(4-sulfophenyl)-2 H-tetrazolium
ANOVA: Analysis of variance
SD: Standard deviation
