# Supplementary material for: Biological, phytochemical and molecular docking characteristics of Laurus nobilis L. fresh leaves essential oil from Palestine
Source: BMC Complement Med Ther. 2024 Jun 8;24:223. doi: 10.1186/s12906-024-04528-9 (PMC11162004; doi:10.1186/s12906-024-04528-9)
Supplement: Supplementary file 1 — Supplementary Material 1 [file 12906_2024_4528_MOESM1_ESM.docx]

**Biological, phytochemical and molecular docking characteristics of *Laurus nobilis* L. fresh leaves essential oil from Palestine**

**Nidal Jaradat ^a,^*, Mohammed Hawash ^a,^*, Mohammed T. Qaoud ^b^, Nawaf Al‑Maharik ^c^, Mohammad Qadi ^d^, Fatimah Hussein ^a^, Linda Issa ^a^, Ahmad Saleh ^a^, Laith Saleh ^a^, Ahmad Jadallah ^a^**

^a^ Department of Pharmacy, Faculty of Medicine and Health Sciences, An-Najah National University, Nablus 00970, Palestine.

^b^ Faculty of Pharmacy, Cyprus International University, Nicosia, Cyprus

^c^ Department of Chemistry, Faculty of Science, An-Najah National University, Nablus 00970, Palestine

^d^ Department of Biomedical Sciences, Faculty of Medicine and Health Sciences, An-Najah National University, Nablus 00970, Palestine.

^*^**Corresponding author**: Nidal Jaradat, Department of Pharmacy, Faculty of Medicine and Health Sciences, An-Najah National University, Nablus, Palestine, Email: nidaljaradat@najah.edu, and **Mohammed Hawash**, Department of Pharmacy, Faculty of Medicine and Health Sciences, An-Najah National University, Nablus, Palestine, orcid.org/0000-0001-5640-9700; Phone: +972569939939; Email: [mohawash@najah.edu](mailto:mohawash@najah.edu).

***GC-MS Assessment method***

The identification of *L. nobilis* essential oil was performed using GC-MS techniques on a Perkin Elmer Clarus 500 gas chromatograph with a Perkin Elmer Clarus 560 mass spectrometer. Separation was achieved using a Perkin Elmer Elite-5 fused-silica capillary column (30 m x 0.25 mm, film thickness 0.25 µm). The column temperature was programmed to increase by 4 °C per minute, starting from 50 °C for 5 min, and reaching 280 °C. Helium was utilized as the carrier gas, maintaining a constant flow rate of 1 mL/min throughout the chromatographic run. For the analysis, 0.2 µl of the tested oil was injected in split mode at a temperature of 250 °C, with a split ratio of 1:50. The mass spectra of the chemical components in the essential oil were compared to the reference spectra from the National Institute of Standards and Technology's MS Data Center, and their Kovats and retention indices were matched against values reported in the literature [1-3].

***Antimicrobial activity***

The antimicrobial activity of *L. nobilis* essential oil was evaluated following a previously established broth microdilution method [4]. The antibacterial effect was tested against five common bacterial species obtained from the American Type Culture Collection (ATCC), including *Escherichia coli, Klebsiella pneumoniae, Proteus mirabilis, Pseudomonas aeruginosa*, and *Staphylococcus aureus*, with ATCC numbers 25922, 13883, 12453, 9027, and 25923, respectively. Additionally, a clinical isolate of Methicillin-resistant *Staphylococcus aureus* (MRSA) from An-Najah National University Hospital was used in the investigation. The anticandidal effect was assessed against *Candida albicans* (ATCC 90028).

Minimal Inhibitory Concentration (MIC) of *L. nobilis* essential oil was determined using the broth microdilution method. The essential oil was initially prepared in DMSO at a concentration of 200 µg/mL. Two-fold serial micro-dilutions were performed ten times (10 wells) in sterile Mueller-Hinton Broth (MHB) for bacterial testing and RPMI for candidal testing. The dilutions were prepared in 96-well plates under aseptic conditions. The wells contained a gradient of *L. nobilis* essential oil concentrations (ranging from 0.1 µg/mL to 50 µg/mL) mixed with the prepared microbial inoculm to a final concentration of 5 * 10 ^5^ CFU/ml in each well for the bacterial strains and 0.5-2.5 * CFU/ml in each well for the candidal strain. Two remaining wells were used as controls, one containing media alone and bacteria or candida for positive growth control, and the other containing media alone for negative growth control. After preparation, the micro-well plates were incubated at 37 °C for 18-24 h for bacterial testing and up to 48 h for *C. albicans* testing.. The minimum concentration of the essential oil that inhibited the growth of the microbe was considered the MIC. Experiments were done in triplicates. To validate the method, Doxycycline (ranging from 0.1 µg/mL to 50 µg/mL) and Ciprofloxacin (ranging from 0.05 µg/mL to 25 µg/mL and from 0.006 µg/mL to 3.125 µg/mL ) were used as controls for antibacterial activity, while Miconazole (ranging from 0.05 µg/mL to 25 µg/mL) served as a control for anticandidal activity [5, 6].

***Porcine pancreatic lipase inhibitory assay***

To assess the anti-obesity activity of *L. nobilis* essential oil, a porcine pancreatic lipase inhibition assay was conducted. Orlistat, a known anti-obesity and anti-lipase drug, was used as a positive control. The porcine pancreatic lipase inhibitory method was based on the protocol described by Zheng et al. with slight modifications [7]. First, a 500 µg/mL stock solution of *L. nobilis* essential oil was prepared by dissolving it in a mixture of dimethyl sulfoxide (DMSO) and methanol (1:9). From this stock solution, five different dilutions (10, 50, 100, 500, and 700 μg/mL) were prepared. A 1 mg/mL stock solution of porcine pancreatic lipase was freshly prepared and dispersed in Tris-HCl buffer. The substrate used for the assay was p-nitrophenyl butyrate (PNPB) obtained from Sigma-Aldrich, Germany, and it was prepared by dissolving 20.9 mg of PNPB in 2 mL of acetonitrile. In the assay, 0.1 mL of porcine pancreatic lipase (1 mg/mL) and 0.2 mL of *L. nobilis* essential oil from each concentration series were combined in five working test tubes. The resulting mixture was adjusted to a total volume of 1 mL using Tris-HCl solution (pH 7.4) and then incubated at 37 °C for 15 min. Afterward, 0.1 mL of the PNPB solution was added to each test tube, and the mixture was incubated for an additional 30 min at 37 °C. The activity of pancreatic lipase was determined by measuring the hydrolysis of PNPB into p-nitrophenolate ions at 410 nm using a UV-Vis spectrophotometer.

The same procedure was repeated for the positive control sample (Orlistat; five different dilutions 10, 50, 100, 500, and 700 μg/mL) obtained from Sigma-Aldrich, Germany. The percentage of inhibition for the anti-lipase activity was determined using the following equation:

$Lipase inhibition (\%)=\frac{AB-Ats}{AB}X100\%$

Where AB is the recorded absorbance of the blank solution, and Ats is the recorded absorbance of the tested sample solution [8].

***α-Amylase inhibition assay***

The α-amylase inhibitory activity of *L. nobilis* essential oil was evaluated using the standard method reported by Nyambe-Silavwe et al. with slight modifications [9]. The essential oil was dissolved in DMSO (Riedel-de-Haen, Germany) and then further diluted with a buffer containing Na_2_HPO_4_/NaH_2_PO_4_ (0.02 M), NaCl (0.006 M) at pH 6.9, to achieve a concentration of 1000 μg/mL. From this solution, a concentration series of 10, 50, 70, 100, and 500 μg/mL was prepared. In the assay, 0.2 mL of porcine pancreatic α-amylase enzyme solution (Sigma-Aldrich, USA) with a concentration of 2 units/mL was mixed with 0.2 mL of the *L. nobilis* essential oil and incubated at 30 °C for 10 min. After that, 0.2 mL of freshly prepared starch solution (1%) was added, and the mixture was incubated for at least 3 min. The reaction was stopped by adding 0.2 mL of dinitrosalicylic acid (DNSA) (AlfaAesar, UK). Subsequently, the mixture was diluted with 5 mL of distilled water and heated in a water bath at 90 °C for 10 min. After cooling to room temperature, the absorbance was measured at 540 nm. A blank was prepared following the same procedure, but instead of *L. nobilis* essential oil, 0.2 mL of the buffer was used. Acarbose (Sigma-Aldrich, USA) was employed as a positive control and underwent the same procedure described above with a concentration series of 10, 50, 70, 100, and 500 μg/mL.

The α-amylase inhibitory activity was calculated using the following equation:

$$\% of \alpha-amylase inhibition =\frac{AB-AS}{AB}X100\%$$

Where Ab represents the absorbance of the blank, and AS is the absorbance of the tested sample or control.

***Antioxidant assay***

To assess the antioxidant potential of *L. nobilis* essential oil, a solution of the oil (1 mg/mL) in methanol was serially diluted with methanol to prepare concentrations of 2, 5, 10, 20, 30, 50, and 80 µg/mL. DPPH (2,2-diphenyl-1-picrylhydrazyl) reagent obtained from Sigma, USA. Subsequently, 1 mL of each concentration was combined with 1 mL of methanol and 1 mL of a 0.002% DPPH solution. The same procedures were repeated for Trolox, which served as a positive control. All the solutions, including *L. nobilis* essential oil and Trolox, were kept in a dark chamber at room temperature for 30 min. Subsequently, their absorbance values were measured at a wavelength of 517 nm using a UV-visible spectrophotometer. The DPPH inhibition potentials of *L. nobilis* essential oil and Trolox were determined using the following equation:

DPPH inhibition (%) = (abs_blank_ - abs_sample_) / abs_blank_ * 100%

where abs_blank_ represents the blank absorbance, and abs_sample_ is the absorbance of the samples. To evaluate the antioxidant half-maximal inhibitory concentration (IC_50_) of *L. nobilis* essential oil and Trolox, the BioDataFit-E1051 program [10] was utilized. This allowed for a quantitative comparison of the antioxidant activities of both substances.

***Cytotoxicity method***

For the culture of breast cancer (MCF-7), skin tumor (B16-F1), and colorectal adenocarcinoma (Caco-2) tumor cells were obtained from ATCC (Rockville, MD, USA). RPMI 1640 medium was utilized as the culture medium. The RPMI 1640 medium was supplemented with 1% L-glutamine, 1% penicillin/streptomycin, and 10% fetal bovine serum. The HeLa cells were cultured in a humidified environment at 37°C with a 5% CO_2_ atmosphere. To perform the experiments, a 96-well plate was used to seed the cells at a density of 5x10^3^ cells/well. After 24 h of cell seeding, the cells were treated with various concentrations (125, 250, 500, and 1000 µg/mL) of the tested essential oil, as well as with Doxorubicin, which served as a positive control. The treatment duration was 48 h. To assess cell viability, the Cell-Titer 96^®^ Aqueous One Solution Cell Proliferation (MTS) bioassay from Promega Corporation (Madison, WI, USA) was employed according to the package recommendations. Following the treatment, 20 μL of MTS solution was added per 100 μL of media in each well, and the plates were incubated at 37 °C for 2 h. After incubation, the absorbance was measured at 490 nm using a UV-Vis spectrophotometer. This measurement allowed for the evaluation of cell viability and the potential effects of the tested essential oil and Doxorubicin on cancer cells [11, 12].

**References**

1. Vinaixa M, Schymanski EL, Neumann S, Navarro M, Salek RM, Yanes O. Mass spectral databases for LC/MS-and GC/MS-based metabolomics: state of the field and future prospects. TrAC Trends in Analytical Chemistry. 2016;78:23-35.

2. Wei X, Koo I, Kim S, Zhang X. Compound identification in GC-MS by simultaneously evaluating the mass spectrum and retention index. Analyst. 2014;139(10):2507-14.

3. Jaradat N, Al-Lahham S, Abualhasan MN, Bakri A, Zaide H, Hammad J, et al. Chemical constituents, antioxidant, cyclooxygenase inhibitor, and cytotoxic activities of Teucrium pruinosum boiss. Essential oil. BioMed research international. 2018;2018.

4. Qadi M, Jaradat N, Al-Lahham S, Ali I, Abualhasan MN, Shraim N, et al. Antibacterial, Anticandidal, Phytochemical, and Biological Evaluations of Pellitory Plant. Biomed Res Int. 2020;2020:<https://doi.org/10.1155/2020/6965306>.

5. Balouiri M, Sadiki M, Ibnsouda SK. Methods for in vitro evaluating antimicrobial activity: A review. J Pharm Anal. 2016;6(2):71-9.

6. Hawash M, Jaradat N, Abualhasan M, Qaoud MT, Joudeh Y, Jaber Z, et al. Molecular docking studies and biological evaluation of isoxazole-carboxamide derivatives as COX inhibitors and antimicrobial agents. 3 Biotech. 2022;12(12):1-16.

7. Zheng C-D, Duan Y-Q, Gao J-M, Ruan Z-G. Screening for anti-lipase properties of 37 traditional Chinese medicinal herbs. Journal of the Chinese Medical Association. 2010;73(6):319-24.

8. Hawash M, Jaradat N, Abualhasan M, Thaher M, Sawalhi R, Younes N, et al. In vitro and in vivo assessment of the antioxidant potential of isoxazole derivatives. Scientific Reports. 2022;12(1):18223.

9. Nyambe-Silavwe H, Villa-Rodriguez JA, Ifie I, Holmes M, Aydin E, Jensen JM, et al. Inhibition of human α-amylase by dietary polyphenols. Journal of Functional Foods. 2015;19:723-32.

10. Jaradat NA, Shawahna R, Hussein F, Al-Lahham S. Analysis of the antioxidant potential in aerial parts of Trigonella arabica and Trigonella berythea grown widely in Palestine: A comparative study. European Journal of Integrative Medicine. 2016;8(5):623-30.

11. Jaradat N, Al-Lahham S, Abualhasan MN, Bakri A, Zaide H, Hammad J, et al. Chemical constituents, antioxidant, cyclooxygenase inhibitor, and cytotoxic activities of *Teucrium pruinosum* boiss. Essential oil. Biomed Res Int. 2018;2018:<https://doi.org/10.1155/2018/4034689>.

12. Hawash M, Eid AM, Jaradat N, Abualhasan M, Amer J, Zaid AN, et al. Synthesis and biological evaluation of benzodioxole derivatives as potential anticancer and antioxidant agents. Heterocycl Comm. 2020;26(1):157-67.
